# Extensive pulmonary involvement in Kaposi sarcoma in a patient with human immunodeficiency virus-acquired immunodeficiency syndrome

**DOI:** 10.1590/0037-8682-0192-2020

**Published:** 2020-11-13

**Authors:** Sildomar Queiroz e Silva, Carlos Henrique Michiles Frank, Taynná Vernalha Rocha Almeida

**Affiliations:** 1 Fundação Centro de Controle de Oncologia do Estado do Amazonas, Manaus, AM, Brasil.; 2 Fundação de Medicina Tropical Dr. Heitor Vieira Dourado, Manaus, AM, Brasil.; 3 Universidade Federal do Amazonas, Faculdade de Medicina, Programa de Pós-graduação em Ciências da Saúde, AM, Brasil.

**FIGURE 1:**
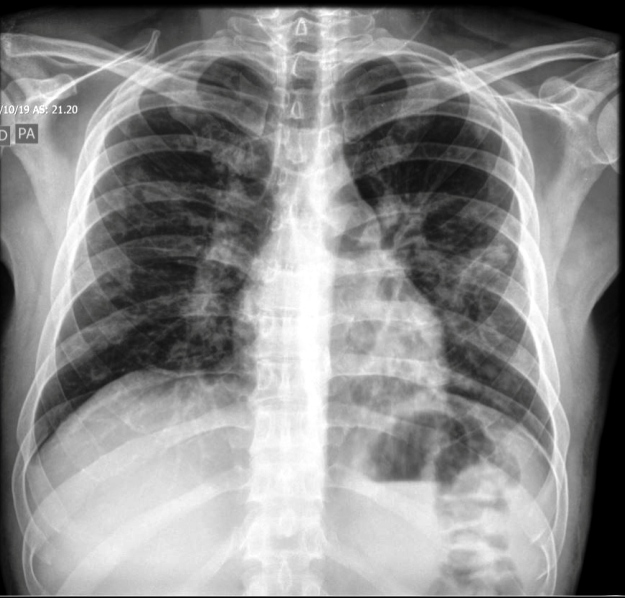
Chest radiography, posteroanterior and lateral views.

A 36-year-old man, HIV-infected for the past eight years, non-adherent to antiretroviral therapy (ART), was admitted to the emergency department with fever, low back pain, asthenia, and dyspnea associated with hyaline-secretive cough. Physical examination showed violaceous lesions on the upper trunk and soft palate. Biopsy confirmed the histopathological diagnosis of Kaposi sarcoma (KS). Chest radiography (Figure 1) showed nodular and reticular parenchyma opacities with a predilection for perihilar, middle, and lower fields, more accentuated on the left lung. 

**FIGURE 2:**
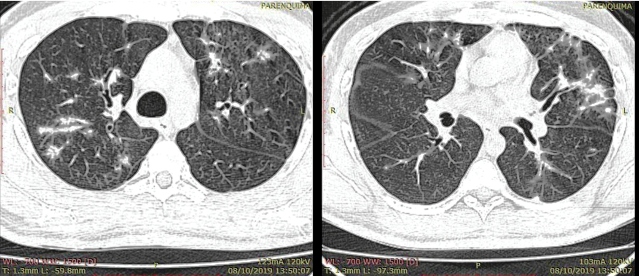
Axial, superior (**left**), and median (**right**) views, 1.3-mm thickness, pulmonary window.

**FIGURE 3:**
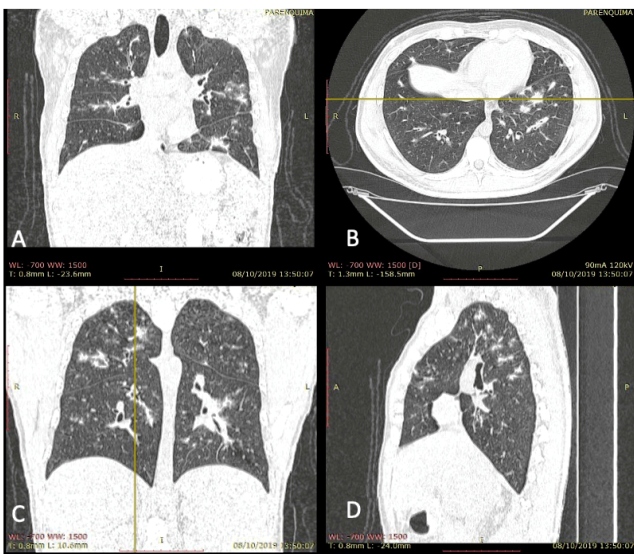
Coronal multiplanar reconstruction (**A, C**), axial view (**B**) and sagittal view (**D**) in a pulmonary window. The yellow line corresponds to the position of each reconstruction.

High-resolution chest computed tomography (CT) (Figures 2 and Figure 3) revealed diffuse ill-defined large nodule opacities with bilateral perilymphatic distribution together with thickening of interlobular septae and peribronchovascular interstitium measuring approximately 1.5 mm. 

These clinical manifestations highlight the potentially aggressive course of KS in HIV-infected patients. linical evaluation with staging of HIV-related KS may determine the future treatment course. Characteristic CT findings in AIDS-related KS include peribronchovascular and interlobular septal thickening, bilateral and symmetric ill-defined nodules in peribronchovascular distribution, fissural nodules, mediastinal adenopathies, and pleural effusions^1^. The propensity for KS to grow in peribronchial and perivascular axial interstitial spaces corroborates the described findings^2^. The introduction of ART has decreased the risk of developing KS^3^; however, KS remains the most common malignancy in HIV-infected patients. 
